# Uneven distribution of human papillomavirus 16 in cervical carcinoma *in situ* and squamous cell carcinoma in older females: A retrospective database study

**DOI:** 10.3892/ol.2014.2347

**Published:** 2014-07-11

**Authors:** SONIA ANDERSSON, MIRIAM MINTS, ULF GYLLENSTEN, MONICA LINDELL, INGER GUSTAVSSON, MATS LAMBE, ERIK WILANDER

**Affiliations:** 1 Department of Women’s and Children’s Health, Division of Obstetrics and Gynecology, Karolinska Institute, Karolinska University Hospital Solna, Stockholm S-171 76, Sweden; 2 Department of Genetics and Pathology, Uppsala University, Uppsala S-751 85, Sweden; 3 Department of Clinical Pathology and Cytology, Uppsala University Hospital and Uppsala University, Uppsala S-751 85, Sweden; 4 Regional Oncologic Centre, Uppsala University Hospital and Uppsala University, Uppsala S-751 85, Sweden

**Keywords:** cervix, carcinoma *in situ*, human papillomavirus 16, prevalence, old age

## Abstract

Human papillomavirus (HPV) 16 is the dominant cofactor in cervical cancer development. The present report investigated the age-specific prevalence of HPV16 in cervical carcinoma *in situ* (CIS) in females attending organised cervical cancer screening. A retrospective observational study was performed based on individual data from two databases. A total of 162 females aged between 20 and 65 years from Uppsala County, Sweden with CIS and an HPV test conducted between 2010 and 2011, preceding or concomitant to CIS diagnosis, were included. Females with cervical squamous cell carcinoma (SCC; n=35) were used for comparison. In total, 96% (n=156) of females with CIS were positive for high-risk HPV; HPV16 was the most prevalent (44.5%), followed by HPV33/52/58 (19.5%), HPV31 (13.1%) and HPV18/45 (9.5%). HPV16 was most frequently detected in females with CIS aged between 20 and 29 years (73.6%) and least frequently detected in those aged between 50 and 65 years (33.3%), with a statistically significant age-specific difference (P=0.001). Among the HPV16-positive females, multiple infections were most frequent in the younger age groups. The prevalence of HPV16 in females with CIS decreased with age, whereas a high prevalence of HPV16 remained in females with SCC. These results may indicate that HPV16 has increased oncogenic potential in older females.

## Introduction

Persistent infection with high-risk human papillomavirus (HPV) types has a critical aetiological role in the development of cervical intraepithelial neoplasia and cervical cancer (1). The progression from low- to high-grade dysplasia and invasive disease is extremely rare in the absence of HPV (2). A recently published meta-analysis included 30,848 cases of invasive cervical cancer; the most frequently found HPV types in descending order were HPV16, 18, 58, 33, 45, 31, 52, 35, 59, 39, 51 and 56, with HPV16 and/or 18 associated with 73% of all cases (3). This is in accordance with the HPV types classified as carcinogenic by the International Agency for Research on Cancer (4).

The eight most frequent HPV types reported by Li *et al* (3) accounted for 91% of 8,977 HPV-positive cervical cancers of epithelial origin in a worldwide retrospective cross-sectional study on HPV type distribution (5). HPV16 and 18 have been reported to be the causal agents in 65–77% of all cervical cancers (5,6), and HPV16 is the most prevalent HPV type in all regions with the exception of Eastern Africa, Japan and Taiwan, where it is the second most prevalent type following HPV52 (5). In a Swedish study, HPV16 was detected in 61% of cervical carcinoma *in situ* (CIS) followed by HPV33/52/58 in 24%, HPV31 in 13% and HPV18/45 in 12% (7).

In countries with organised cervical cancer screening programmes, mortality from cervical cancer occurs most commonly among older females. Despite screening and protocols for the treatment of cervical intraepithelial neoplasia, which have existed for several decades, invasive cervical cancer remains common in females aged ≥50 years. In 2006, >60% of cervical squamous cell carcinomas (SCCs) in Sweden occurred in postmenopausal females aged ≥50 years (8) and 86% of all cervical cancer-related mortalities occurred in females belonging to older age groups (9,10). It has been shown that HPV testing is three times as sensitive as cytology to detect CIN2 or worse in females aged between 50 and 65 years (11). However, diagnostic difficulties remain, as knowledge with regard to the prevalence of HPV infection in this age group is limited. For this reason, the understanding of the correlation between high-risk HPV infection and cervical cancer in older females is of significant value (12).

The purpose of this study was to identify the prevalence of HPV16 in females with CIS in the population-based organised cervical screening programme in Uppsala County, Sweden, and to describe the age-specific prevalence of multiple HPV infections, with a focus on older females. The prevalence of HPV16 in females with CIS was also compared with that in females with SCC.

## Patients and methods

### Organised cervical cancer screening programme in Uppsala County, Sweden

The population-based organised cervical screening programme in Uppsala County, Sweden screens females aged between 25 and 60 years. Within the framework of the programme, ~70,000 females are screened by conventional cytology each year by midwives in family planning centres. Females with abnormal cytological findings, using atypical squamous cells of undetermined significance (ASCUS) as a cut-off, are referred for further investigation. Females with low-grade cytological abnormalities [such as ASCUS and low-grade squamous intraepithelial lesions (SIL)] undergo supplementary HPV testing and have a new cytological sample taken following a mean follow-up time of approximately three months, at which time two cervical brushes (MedScand, Malmö, Sweden) are used to collect cell samples from the ecto- and endocervix. One brush is spread on a slide for Papanicolaou staining, while the other is applied to a FTA elute card (filter paper matrix; cat. no. WB120411; Whatman, Inc., Clifton, NJ, USA) (13,14). HPV detection is performed using an hp*VIR* multiplex real-time polymerase chain reaction (PCR) assay. Females with persistent ASCUS/low-grade SIL and high-risk HPV infection at the three-month follow-up, as well as females with high-grade SIL, are referred for a complete clinical work-up, including a pelvic examination, colposcopy with directed biopsies of suspicious areas and repeat cytology.

The SymPathy database (Tieto AB, Malmö, Sweden) contains information on results obtained at the Department of Clinical Pathology and Cytology of Uppsala University Hospital (Uppsala, Sweden). Data therein are identified by specific topography and morphology codes (Systematized, Nomenclature Of Medicine; College of American Pathologists, Skokie, IL, USA). The database of the organised population-based cervical cancer screening programme of Uppsala County, Sweden includes cytological, HPV and histological results from all laboratories in the county. The cytological and histological samples from the screening programme are stored at the Department of Clinical Pathology and Cytology of Uppsala University Hospital (Uppsala, Sweden), where all cytological, HPV and histological testing for the screening programme is performed. Detailed descriptions of the database searches for HPV status and HPV screening analysis results have been published (14).

Briefly, a 3-mm Ø Harris micro punch (GE Healthcare, Little Chalfont, UK) is used to excise sections from the FTA cards using the BSD 600 (BSD Robotics, Queensland, Australia) semi-automatic punch robot. Four punches from each card are then transferred to a single well in a 96-well plate and washed by vortexing three times for 5 sec in 200 μl distilled water. The water is then carefully removed using a pipette. DNA elution is performed in 50 μl of distilled water at 95°C for 30 min in a heating block (with a heated lid). A total of 3 μl of the DNA extract is used as a template in each real-time PCR (14). HPV typing is performed as previously described, using a real-time PCR assay (13), which detects and quantifies a human single copy gene [housekeeping gene; homo sapiens hydroxymethylbilane synthase (HMBS); GenBank accession no. M95623.1] and the following HPV types, partly in groups: HPV16, 18/45, 31, 33/52/58, 35, 39, 51, 56 and 59. In order to determine if a sample contains a sufficient amount of material for HPV testing, a threshold of 10 copies of the nuclear single copy gene per PCR is used, based on the HMBS analysis. In addition, the sample has to contain a minimum of 10 HPV copies to render a positive HPV result (14).

### Study population

Females aged between 20 and 65 years with a histopathological diagnosis of CIS determined between 2010 and 2011 were identified in the Sympathy database. Previous cytological, HPV and histological results for these females were taken from the database of the cervical cancer screening programme of Uppsala County, Sweden. The study population was then restricted to females with a preceding or concomitantly performed HPV test, separated by age group and further restricted to females who tested positive for HPV.

Older females with SCC were used for comparison. Females aged between 50 and 95 years with a diagnosis of SCC determined between 2008 and 2011, and a preceding or concomitant HPV test were identified and included in the study.

### Statistical analysis

Data were analysed using the Statistica 6.1 software (Statsoft, Inc., Tulsa, OK, USA). Pearson’s and Yates’ corrected χ^2^ (for n<5) were used to compare proportions.

### Ethics statement

The present study was approved by the Uppsala University Ethics Review Board (Uppsala, Sweden), which determined that informed consent from the participants was not required, and was conducted according to the principles expressed in the Declaration of Helsinki.

## Results

### HPV types in CIS

Of the 205 females with CIS identified in the database, 162 (79%) had a previous or concomitant HPV test recorded in the database of the organised cervical cancer screening programme. Of those, 156 (96%) tested positive for HPV and were included in the final analyses. In addition, 35 females with SCC were also included for comparison. The mean age of females with CIS was 37 years, (median, 37; range, 20–65 years) and the mean age of females with SCC was 66 years (median, 62; range 50–95 years).

**Figure 1:**
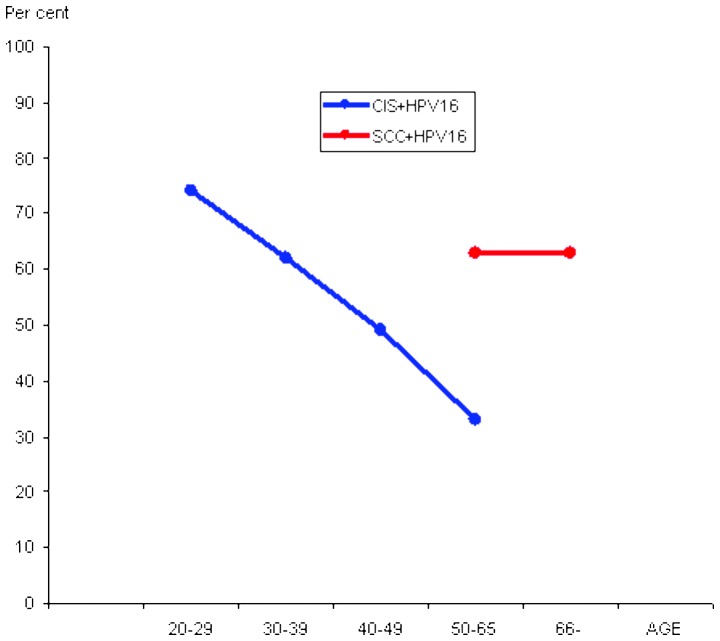
Prevalence of HPV16 (%) in HPV-positive females with cervical CIS (CIS + HPV16; n=156) in relation to age, and in older females (age range, 50–95 years) with SCC (SCC + HPV16; n=30). HPV, human papillomavirus; CIS, carcinoma *in situ*; SCC, squamous cell carcinoma.

HPV16 was the most common high-risk HPV type in this study population (n=89; 44.5%). Age-specific HPV16 prevalence ranged from 73.6% (28/38) in females aged between 20 and 29 years to 33.3% (7/21) in females aged between 50 and 65 years (P=0.001; χ^2^). Whereas HPV18/45 was only identified in 19 (9.5%) females of the study population (P<0.001 vs. HPV16; χ^2^). HPV33/52/58 and HPV31 were also more common than HPV18. HPV33/52/58 was found in 13.1% of females aged between 20 and 29 years and in 30% of females aged ≥50 years, and the HPV33/52/58 types were as common as HPV16 in females aged ≥50 years (30.4%) (Tables I and II and Fig. 1). The importance of high-risk HPV types other than HPV16 varied somewhat with age. In general, HPV33/52/58 and HPV31 were more frequent among females aged between 20 and 49 years (25.0 and 17.0%, respectively) than HPV18/45, which occurred in 12.0% of the study population (Table I).

### Multiple HPV types

Multiple infections were common in this study population, but more so among the younger females. Overall, 38.9% of females harboured more than one HPV type, and 26.3% of females aged between 20 and 29 years harboured more than one high-risk HPV type, compared with 9.6% of females aged between 30 and 39 years, and 3.0% of females ages ≥40 years. In HPV16-positive females, multiple infections were also more prevalent in younger females; 35.6% of females aged between 20 and 29 years, compared with ~7.0% in females aged ≥40 years (Table II and Fig. 1).

### HPV types in SCC

In total, 30 (85.7%) females with SCC were positive for high-risk HPV types. HPV16 predominated and occurred in 19 (63.3%) females with SCC, followed by HPV18/45 in six (20.0%) females. However, multiple HPV infections were not recorded in any females with SCC (Table III).

The biopsies of the six females with CIS and a negative HPV test were re-evaluated, but the diagnosis was not altered. The biopsies of the five females with SCC and a negative HPV test were also re-evaluated, but in the majority of cases the biopsies were small, and it was not possible to determine whether the tumour was of cervical origin, or constituted local metastases of a tumour with another primary site.

## Discussion

This study provides an overview of the HPV type distribution, and the prevalence of multiple infections encountered among females of all ages with CIS detected in a population- and cytology-based cervical cancer screening programme, as well as among older females with SCC. The prevalence of HPV16 was found to vary with age in females with CIS, with a significant decreasing prevalence in older females. Other studies have identified the same pattern, showing HPV16 as the dominant oncogenic HPV type in CIS (7,15). In females with CIS aged between 25 and 49 years, 61% were HPV16-positive, a figure identical to that of a previous study (7). However, by separating females into age groups and including older females aged between 50 and 65 years, a clear trend was observed, namely a markedly significant (P<0.001) decrease in HPV16 positivity to 33% among females with CIS in the oldest age group.

A combination of organised and opportunistic screening has reduced the incidence of SCC substantially during the last decades in Sweden (16,17). However, the sensitivity of cytological screening is lower in older females (18,19), and it has been indicated that in spite of a high incidence of cervical cancer in older females, the value of screening in older age groups is questionable (18). In addition, the sensitivity of cytology testing has been reported to be lower than HPV testing in older females (11,19). Consistent with this finding, countries with organised cervical cancer screening programmes have a higher occurrence of cervical cancer-related mortality among females aged ≥50 years (10). For that reason, the correlation between high-risk HPV infection and cervical cancer in older females deserves attention, and has been the object of increased interest (11,19). A higher risk of premalignant and malignant cervical alterations in younger, compared with older, HPV16-positive females has been reported (20). The current study observed a decreasing importance of HPV16 infection with increasing age in females with CIS. Conversely, the number of older females with SCC harbouring HPV16 was unaltered at ~63%. This suggested that the discrepancy between the number of HPV-positive premalignant and malignant cervical lesions increases to a significant degree with age, however, the reason for this is unknown. It has been reported that a polymorphism of the HPV16 E6 gene is common and special attention has been directed to a L83V mutation in the HPV16 E6 gene (21,22) that may affect the oncogenic potential of the virus. Further studies are required to identify the transformation activity of various E6 proteins of high-risk HPV types.

In conclusion, HPV testing exhibits a considerably higher sensitivity to detect CIS compared with conventional cytology (23), particularly in older females (11,19). The prevalence of high-risk HPV infection decreases with age, and in older females is ~6% (12), meaning that the specificity of primary high-risk HPV testing increases with age. Furthermore, this specificity can be refined by short-time repeat HPV testing to identify females with persistent infection, who are at higher risk of cervical cancer development (24). When launching primary high-risk HPV testing in older females, the oncogenic potential of various high-risk HPV types, including the prototypes and their variants, may be of value (25).

## Figures and Tables

**Table I:** Distribution of high-risk HPV types in 156 females with cervical carcinoma *in situ*.

Age, years	HPV16, n	HPV18/45, n	HPV33/52/58, n	HPV31, n	Others, n	Total, n
20–29	28	6	8	8	11	61
30–39	32	5	13	8	8	66
40–49	22	5	11	7	5	50
50–65	7	3	7	3	3	23
Total (%)	89 (44.5)	19 (9.5)	39 (19.5)	26 (13.0)	27 (13.5)	200^a^

a Number of identified HPV types exceeded the number of females examined due to the occurrence of multiple HPV infections.

HPV, human papillomavirus.

**Table II:** Occurrence of HPV16 infection in 156 females with CIS and of multiple HPV infections by age.

Age, years	CIS	HPV16, n (%)	HPV16 with multiple infections, n (%)
20–29	38	28 (73.6)	10 (35.7)
30–39	52	32 (61.5)	5 (15.6)
40–49	45	22 (48.8)	1 (2.2)
50–65	21	7 (33.3)	1 (4.8)
Total	156	89 (57.0)	17 (19.1)

HPV, human papillomavirus; CIS, cervical carcinoma *in situ.*

**Table III:** Prevalence of high-risk HPV types in cervical squamous cell carcinoma of 30 females of ≥50 years of age (range, 50–95 years) between 2008 and 2011.

HPV type	n (%)
16	19 (63.3)
18/45	6 (20.0)
31	1 (3.33)
33/52/58	1 (3.33)
35	1 (3.33)
36	1 (3.33)
59	1 (3.33)
Total	30 (100)^a^

a No multiple infections.

HPV, human papillomavirus.
